# Human Milk Oligosaccharides: The Journey Ahead

**DOI:** 10.1155/2019/2390240

**Published:** 2019-08-04

**Authors:** Chandan Ray, Joshi A. Kerketta, Subhash Rao, Snehal Patel, Shantanu Dutt, Kamal Arora, Femitha Pournami, Phani Bhushan

**Affiliations:** ^1^Bhagirathi Neotia Woman and Child Care Center, 2 Rawdon Street, Park street Area, Kolkata 700017, West Bengal, India; ^2^Healthworld Hospitals, C-49, Commercial Area, Opp. ESIC, Sub-Regional Office, Near Gandhi More, City Centre, Durgapur 713216, West Bengal, India; ^3^Fortis Hiranandani Hospital, Mini Sea Shore Road, Sector 10, Vashi, Navi Mumbai 400703, Maharashtra, India; ^4^Smt. NHL Municipal Medical College, Pritan Rai Cross Road, Ellise Bridge, Paldi, Ahmedabad 380006, Gujarat, India; ^5^Dr. Dutt Children Hospital & Nursing Home, Parker College Rd, Budh Bazaar, Moradabad 244001, Uttar Pradesh, India; ^6^Dayanand Medical College & Hospital, Tagore Nagar, Civil Lines, Ludhiana 141001, Punjab, India; ^7^Kerala Institute of Medical Science, P.B. No. 1, Anayara P.O., Trivandrum 695029, Kerala, India; ^8^Columbia Asia Referral Hospital Yeshwanthpur, 26/4, Brigade Gateway, Beside Metro Cash and Carry West, Malleshwaram, Bengaluru 560055, Karnataka, India

## Abstract

Breast milk is a complex biological fluid that is rich in nutrients and bioactive agents that support the healthy growth and development of the newborns. Human milk oligosaccharides (HMOs) are unconjugated glycans that constitute an important component of the protection conferred by breast milk on the neonate. HMOs may act locally on the neonatal intestine by acting as signalling molecules and directly interacting with the host cells. Although fucosylated and sialylated HMOs have little nutritional value, they exert important prebiotic as well as immunomodulatory effects on the infant gut. However, there is heterogeneity in the quantity and quality of HMOs in breast milk produced by mothers under influence of the genetic and environmental factors. This review encompasses the salient aspects of HMOs such as composition, function, structural diversity, and functional impact on the growth and survival of newborns. In this review, the current knowledge on HMOs is contextualised to discuss the gaps in scientific understanding and the avenues for future research.

## 1. Introduction

Human milk is considered as the “gold standard” of infant nutrition during the first few months of human life. The composition of human milk is unique and it is adapted for the infant's immature digestive and immune systems. Human milk protects infants from infection and inflammation, promotes development of immunity, and facilitates organ maturation [1]. Human milk oligosaccharides (HMOs) are a part of functional ingredients of the breast milk. HMOs are complex glycans that are found in high concentrations and with unique structural diversity. HMOs are the third most abundant components of milk after lactose and lipids [2].

## 2. Structure of HMOs

**Figure 1:**
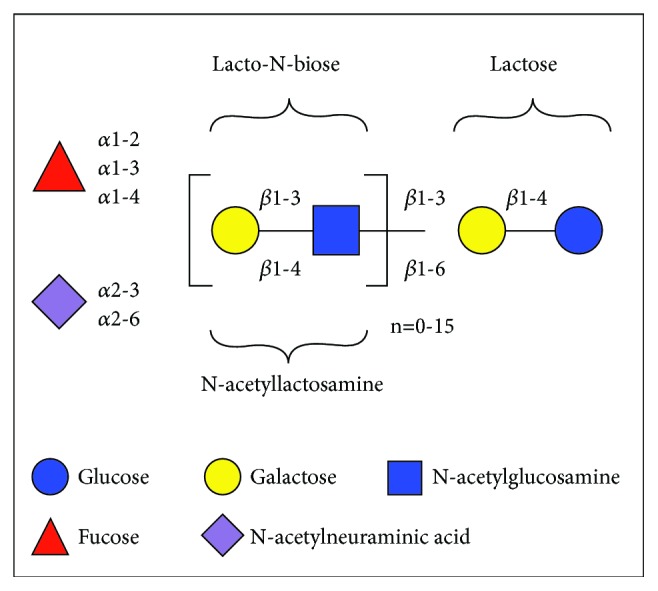
Structural blueprint of HMOs. The glucose, galactose,* N*-acetylglucosamine, fucose, and N-acetylneuraminic acid molecules are the building blocks for HMOs.

Although HMOs are of various types and carry out diverse functions, they have a basic structural blueprint (Figure 1). Five monosaccharides represent the building blocks of HMOs, namely, glucose, galactose,* N*-acetylglucosamine, fucose, and* N*-acetylneuraminic acid [3]. A proportion of 35-50% of fucosylated, 12-14% of sialylated, and 42-55% of nonfucosylated neutral HMOs has been reported in term breast milk [4]. In colostrum, transitional and mature milk HMO concentrations may exceed up to 20 g/L and drop to 5-12 g/L [5].

Although the synthetic oligosaccharides such as galactooligosaccharides (GOS) and fructooligosaccharides (FOS) and pectin-derived acidic oligosaccharide (pAOS) have been introduced into the infant formula, they are structurally different from the HMOs. Fructose or its polymers as well as galacturonic acid and its homo- or heteropolymers are not found in human milk. Fucosylated and sialylated oligosaccharides are important HMOs that are yet to be synthesized [2]. However, the European Food Safety Authority (EFSA) and the U.S. Food and Drug Administration (FDA) have independently confirmed the safety of 2′-fucosyllactose (2′-FL) and lacto-N-neotetraose (LNnT) for administration to babies through infant formulas.

### 2.1. Types of HMOs

HMOs are classified into three types [6]:

- Neutral or fucosylated HMOs: these HMOs contain fucose at the terminal position. Examples: 2'-fucosyllactose (2'-FL) and lactodifucopentose.
- Neutral N-containing or nonfucosylated HMOs: these HMOs contain N-acetylglucosamine at the terminal end. Example: lacto-N-tetraose.
- Acidic or sialylated HMOs: these HMOs contain sialic acid at the terminal end. Example: 2'-sialyllactose.

## 3. Variation in HMOs

The total HMO concentration decreases within the first 3 months, but the level of some HMOs may increase [5]. More than a hundred different HMOs have been identified so far, but not every woman synthesizes the same set of oligosaccharides due to the role of various factors [2]. It is of clinical interest to understand how the multifactorial web of causation may influence the quantity and quality of HMOs in the breast milk.

### 3.1. Secretor Status and Lewis Blood Group

Significant biological variation is evident in specific HMO structures such as 2′-FL and lacto-N-fucopentaose I and II (LNFP I and LNFP II) in mature term breast milk. The variation in *α*1-2-fucosylated HMOs is dependent on the mother's genes that encode for fucosyltransferase (FUT) enzymes, which in turn are determined by the secretor (Se) status and Lewis (Le) blood group [7]. Abundant levels of 2'FL, LNFP I, and other *α*1-2-fucosylated HMOs are present in the milk of secretor mothers. However, fucosyltransferase 2 (FUT2) enzyme and *α*1-2-fucosylated HMOs are absent in nonsecretors [3]. The LIFE cohort study of German mothers demonstrated that 2'FL together with LNFP I and II are reliable proxies to define FUT 2 and 3 status, respectively. The changes in maternal FUT polymorphisms were the determinants of HMO variation with time [8].

### 3.2. Duration of Lactation

The HMOs vary in structure and composition over the course of lactation. A cross-sectional study of Chinese mothers reported that they produced unique types of HMOs during the different stages of lactation, independent of the mode of delivery and geographical location [9]. The overall HMO concentration decreases across lactation, but the direction of the change varies among specific HMOs. A study of HMOs in milk samples of German women from days 3 to 90 postpartum indicated that the levels of 2'FL and LNFP I reduced with increasing duration of lactation, whereas levels of 3-fucosyllactose (3FL) increased in Se+ and Le+ women [10]. Therefore, the activity of Se- and Le-independent fucosyltransferases (FUT3, 4, 5, 6, 7, or 9) may increase across lactation [11]. However, the major small neutral HMOs and their isomers varied the least across lactation [12].

### 3.3. Gestation Period

The total HMO concentration in milk of women who deliver preterm is greater than of those who deliver at term [13]. A mass spectrometric analysis of the fucosylation and sialylation in HMOs in serial milk specimens of women delivering preterm and term indicated that lacto-N-tetraose (LNT) was more abundant and more variable in preterm milk than in term milk. Moreover, the concentration of 2′-FL was not consistent across lactation for mothers with preterm delivery [14]. Thus, the fucosylation of HMOs in preterm milk may be unpredictable [11].

### 3.4. Maternal Health

HMO concentrations were significantly lower in women with a body mass index (BMI) of 14 to 18 than in women with a BMI of 24 to 28 [3]. The ATLAS cohort study of European mothers indicated that the prepregnancy BMI and gestational weight gain may influence individual HMO levels. However, the effects of these on the HMO concentrations were small [15]. Gestational diabetes mellitus did not impact the level and composition of total HMOs [16].

## 4. Function of HMOs

**Figure 2:**
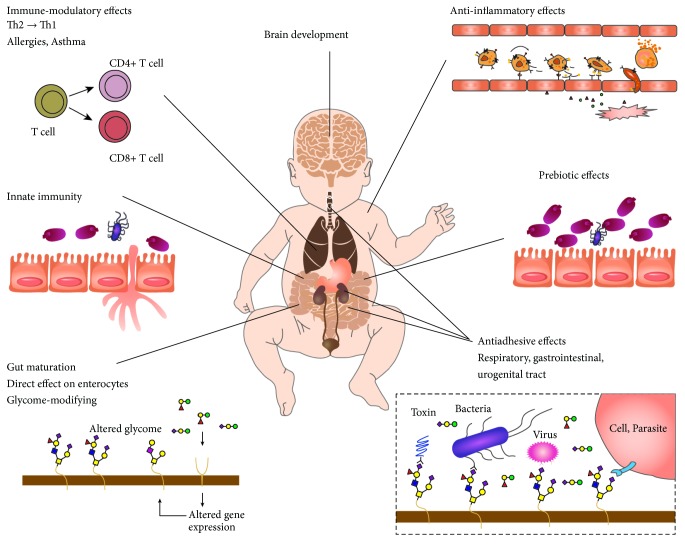
Potential benefits for breast-fed neonates. HMOs may serve as prebiotics, immune-modulators, and signalling molecules to enhance the gut immunity of newborns.

The multifarious functions of HMOs are as follows: (i) they act as prebiotics and stimulate the colonization of beneficial microbes, (ii) they exert direct defence mechanisms against pathogens and protect infants from infections, (iii) they act as signalling molecules and interact directly with the host cells, (iv) they act as anti-inflammatory and immune-modulators, and (v) they act as nutrients for neurological development of infants (Figure 2) [5].

### 4.1. Role of HMOs as Prebiotics

**Figure 3:**
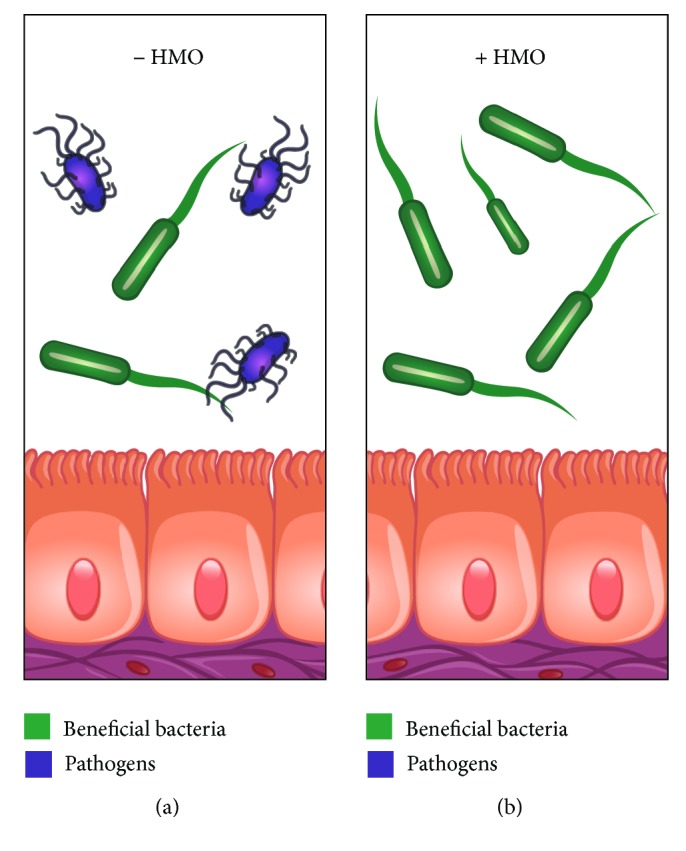
Type of gut microbiota in (a) absence of HMOs and (b) presence of HMOs. The growth of beneficial bifidobacteria is favoured by HMOs over that of pathogens in the gut.

The complex oligosaccharide mixture within HMOs attracts both mutualistic mucus adapted species and HMO-adapted bifidobacteria to the infant intestine [17]. Several in vitro studies have reported that bifidobacterial species can grow on HMOs [5]. This type of commensal feeding is one of the key factors influencing gut microbiota composition and development of immunity in infants [18]. Most of the HMOs are unabsorbed into the gut, where certain bacteria ferment them into short chain fatty acids creating an acidic environment. The low pH environment in the gut favours the growth of other strains of beneficial bifidobacteria [19]. HMOs do not allow the growth of potentially harmful bacteria by favouring the growth of* Bifidobacterium* species (Figure 3) [2]. Evidence suggests that breast-fed infants have a higher abundance of beneficial bifidobacteria compared with formula-fed infants [18].

The genomic study of* Bifidobacterium longum* subsp.* infantis* indicated that it is adapted for utilisation of the HMOs in the infant gut. The conservation of gene clusters in multiple isolates confirms the genomic mechanisms for this infant associated phylotype [20].* B. longum *subsp*. infantis* equally incorporates type 1 and type 2 HMOs, while other bifidobacterial species have preferential use for type 1 HMOs. The predominance of type 1 structures found in HMOs and the conservation of galacto-N-biose (GNB)/lacto-N-biose (LNB) pathway in bifidobacteria indicate that they have coevolved with humans [21]. Mass spectrometry-based glycoprofiling of the HMO consumption behavior revealed a specific preference for fucosylated oligosaccharides by* B. longum* subsp.* infantis* and* Bacteroides vulgatus* [22]. Small-mass HMOs, mainly secreted in the colostrum and during the first month of lactation, are preferred by* B. longum biovar infantis* ATCC 15697, which possesses fucosidase and sialidase activities [23]. A randomized controlled trial of healthy term infants indicated that formulae supplemented with two HMOs, 2'FL and LNnT, may shift the gut microbiota composition towards that of breast-fed infants [24, 25]. Infants fed by nonsecretor mothers are late in the establishment of a bifidobacteria-laden gut ecosystem [26]. Child and mothers' secretor status have an impact on children's microbiota composition at 2 to 3 years of age [27].

### 4.2. Antiadhesive Antimicrobial Properties of HMOs

**Figure 4:**
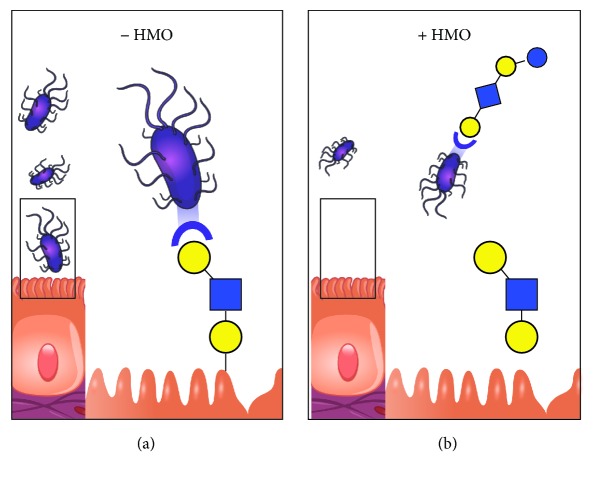
Adhesion of pathogens to gut wall in (a) absence of HMOs and (b) presence of HMOs. The adhesion of the pathogens to the gut wall is prevented by HMOs that serve as decoy.

HMOs are the major constituent of an innate immune system whereby the human milk protects the infant from enteric and other pathogens [28–30]. HMOs prevent the attachment of pathogens by serving as soluble glycan receptor decoys (Figure 4). HMOs directly reduce microbial infections by acting as antiadhesive antimicrobials and indirectly keep the pathogens in check by providing competitive advantage to nonpathogenic commensals [2]. HMOs may mimic structures of viral receptors and block adherence to target cells, thus preventing infection [31].

The sialylated fraction of HMOs showed a strong inhibitory capacity for hemagglutination mediated by enterotoxigenic* Escherichia coli* (ETEC) and uropathogenic* Escherichia coli* (UPEC) [32]. HMOs show a high efficiency in blocking the lectins that contribute to virulence of* Pseudomonas aeruginosa* [33]. Inhibition of the attachment of* Streptococcus pneumoniae* and* Haemophilus influenzae* to human pharyngeal or buccal epithelial cells is attributed to HMO fraction of the breast milk [34]. Jantscher-Krenn et al. demonstrated through* in vitro* studies that HMOs reduced the attachment and cytotoxicity of* Entamoeba histolytica* to intestinal epithelial cells [35]. Specific HMOs inhibit the binding of* Campylobacter jejuni* to the intestinal H-2 antigens [36]. The sialyl Lewis X and B receptors on porcine milk proteins prevent the colonisation of* Helicobacter pylori *[37]. The physiological concentrations of HMOs significantly reduce the gp120 binding of Human Immunodeficiency Virus (HIV)-1 virus to the dendritic cell-specific ICAM3-grabbing nonintegrin (DC-SIGN) on human dendritic cells by more than 80% [38]. DC-SIGN is selective in its recognition of specific types of fucosylated glycans and subsets of oligomannose- and complex-type N-glycans among HMOs [39]. Lewis X motif present in human milk can bind to DC-SIGN and thereby prevent the capture and subsequent transfer of HIV-1 to CD4+ T lymphocytes [40].

### 4.3. Effect of HMOs on Intestinal Epithelial Cells

**Figure 5:**
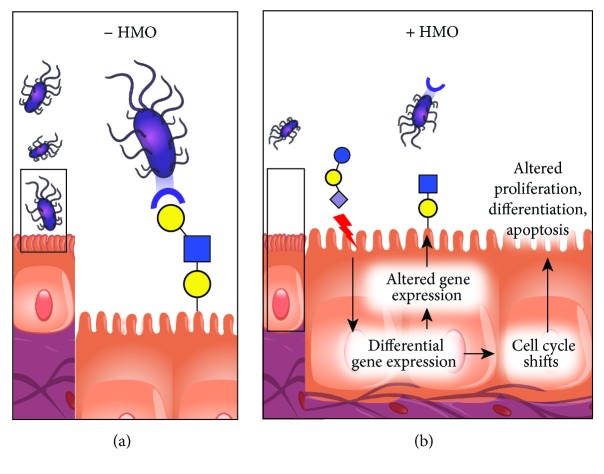
Gut epithelial cells in (a) absence of HMOs and (b) presence of HMOs. The interaction with HMOs leads to altered gene expression and growth of the intestinal epithelial cells.

The interaction of HMOs with pathogenic microbes prevents their attachment to the intestinal epithelial cells [7]. HMOs act directly on the intestinal epithelial cells and modulate their gene expression leading to changes in cell surface glycans and other responses (Figure 5). A study demonstrated that HMOs reduce cell growth and initiate differentiation and apoptosis in cultured human intestinal epithelial cells [2].

HMOs, especially disialyllacto-N-tetraose, contribute to protection from necrotising enterocolitis [41]. HMOs are effective at influencing various stages of intestinal epithelial cells during the gastrointestinal development in vitro [42]. HMOs inhibited intestinal cell proliferation and altered cell cycle dynamics by affecting corresponding regulator genes and mitogen-activated protein kinase signaling [43]. Acidic HMOs serve contribute to the lower incidence of inflammatory diseases such as necrotizing enterocolitis in breast-fed infants [44].

### 4.4. Impact of HMOs on Immune Cells

**Figure 6:**
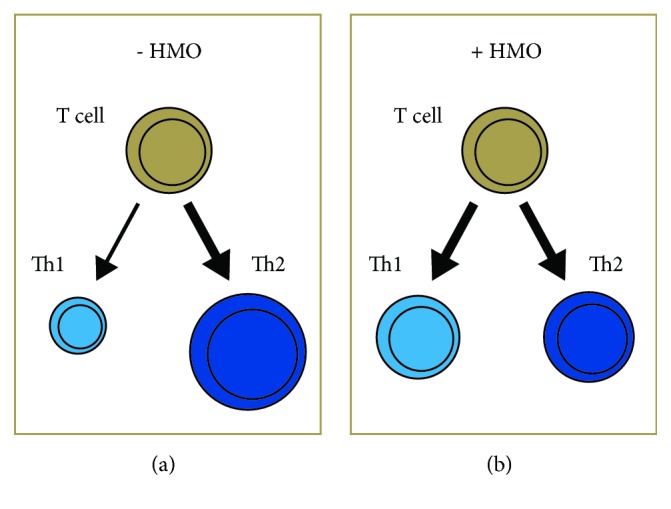
T-cell response in (a) absence of HMOs and (b) presence of HMOs. The modulation of the immune system by the HMOs leads to a more balanced T-cell response.

HMOs exert anti-inflammatory effect by reducing the platelet-neutrophil complex formation that contributes to a reduction in the neutrophil beta-2 integrin expression [44]. Specific HMOs serve as anti-inflammatory components by inhibiting the leukocyte rolling and adhesion to endothelial cells under dynamic conditions [45]. Acidic HMOs affect cytokine production and activation of cord blood derived T cells in vitro [46]. HMOs help in developing the immune system, potentially leading to a more balanced Th1/Th2 response (Figure 6).

Acidic HMOs may modulate postnatal allergen-specific immune responses by suppression of Th-2 type responses in atopy-prone individuals [47]. A randomized controlled trial reported that the healthy term infants fed formulas supplemented with 2'-FL reduced the cytokine levels similar to those of a breast-fed reference group [48]. Infants born by C-section and having a high hereditary risk for allergies might have a lower risk to manifest IgE-associated eczema at 2 years when fed breast milk with FUT2-dependent HMOs [49].

### 4.5. Growth and Survival by HMOs

A randomized controlled study of healthy term infants reported that the formulas supplemented with 2'-FL were well tolerated, and 2'-FL absorption profiles were similar to those of breast-fed infants. There were no significant differences in weight, length, and head circumference between infants fed human milk or 64.3 kcal/dL formulas from birth to 4 months of age [50]. Another randomized controlled trial of healthy infants indicated that the formula with 2'FL and LNnT is safe, well-tolerated and supports age appropriate growth [51]. A cohort study of healthy mothers and infant pairs reported that the relatively substantial variation in HMOs between the high and low 2'FL clusters do not impact infant growth of either sex up to 4 months of age [52]. A study of Gambian mothers and infants indicated that the HMO, 3′-sialyllactose, was a good indicator of infant weight-for-age [53]. A study of Zambian HIV+ mothers and infants demonstrated that breastfeeding was protective against mortality only in HIV exposed, uninfected children with high concentrations of fucosylated HMOs [54].

## 5. The Journey Ahead

HMOs represent the next frontier in neonatal nutrition as they constitute a major component of the immune-protection conferred by breast milk upon vulnerable infants. Progress in clinical research has deemed supplementary provision of HMOs an attractive alternative for newborns who cannot be breast-fed. Although there have been significant breakthroughs in our knowledge about the HMOs, there are many key questions that need to be answered. Basic science should dictate the specific choice of HMOs and clinical data should justify the need for supplementing the infant formulas with them. Clinical research could be directed towards addressing pertinent queries such as which are the specific HMOs, in what quantity and for how long should they be administered. Basic research has hitherto laid a firm groundwork for clinical research on HMOs, which in turn has engendered new questions about their clinical implications.

“*Science never solves a problem without creating ten more.*”* George Bernard Shaw*

## Abbreviations

HMOs: Human milk oligosaccharides
GOS: Galactooligosaccharides
FOS: Fructooligosaccharides
pAOS: Pectin-derived acidic oligosaccharide
EFSA: European Food Safety Authority
USFDA: United States Food and Drug Administration
2'-FL: 2′-fucosyllactose
LNnT: Lacto-N-neotetraose
LNFP: Lacto-N-fucopentaose
FUT: Fucosyltransferase
Se: Secretor
Le: Lewis
3FL: 3-fucosyllactose
LNT: Lacto-N-tetraose
GNB: Galacto-N-biose
LNB: Lacto-N-biose
ETEC: Enterotoxigenic* Escherichia coli*
UPEC: Uropathogenic* Escherichia coli*.

## References

[1] Ballard O., Morrow A. L. (2013). Human Milk Composition: nutrients and bioactive Factors. *Pediatric Clinics of North America*.

[2] Bode L. (2012). Human milk oligosaccharides: every baby needs a sugar mama. *Glycobiology*.

[3] Bode L., Jantscher-Krenn E. (2012). Structure-function relationships of human milk oligosaccharides. *Advances in Nutrition*.

[4] Totten S. M., Zivkovic A. M., Wu S. (2012). Comprehensive profiles of human milk oligosaccharides yield highly sensitive and specific markers for determining secretor status in lactating mothers. *Journal of Proteome Research*.

[5] Jantscher-Krenn E., Bode L. (2012). Human milk oligosaccharides and their potential benefits for the breast-fed neonate. *Minerva Pediatrica*.

[6] Plaza-Díaz J., Fontana L., Gil A. (2018). Human milk oligosaccharides and immune system development. *Nutrients*.

[7] Kunz C., Rudloff S., Baier W., Klein N., Strobel S. (2000). Oligosaccharides in human milk: structural, functional, and metabolic aspects. *Annual Review of Nutrition*.

[8] Binia A., Lefebvre G., Charpagne A. (2018). Time of lactation and maternal fucosyltransferase polymorphisms are main determinants of human milk oligosaccharide variation. ESPGHAN 51 Annual Meeting. *Journal of Pediatric Gastroenterology and Nutrition*.

[9] Austin S., De Castro C., Bénet T. (2016). Temporal change of the content of 10 oligosaccharides in the milk of chinese urban mothers. *Nutrients*.

[10] Thurl S., Munzert M., Henker J. (2010). Variation of human milk oligosaccharides in relation to milk groups and lactational periods. *British Journal of Nutrition*.

[11] Smilowitz J. T., Lebrilla C. B., Mills D. A., German J. B., Freeman S. L. (2014). Breast milk oligosaccharides: structure-function relationships in the neonate. *Annual Review of Nutrition*.

[12] Niñonuevo M. R., Perkins P. D., Francis J. (2008). Daily variations in oligosaccharides of human milk determined by microfluidic chips and mass spectrometry. *Journal of Agricultural and Food Chemistry*.

[13] Gabrielli O., Zampini L., Galeazzi T. (2011). Preterm milk oligosaccharides during the first month of lactation. *Pediatrics*.

[14] De Leoz M. L., Gaerlan S. C., Strum J. S. (2012). Lacto-N-tetraose, fucosylation, and secretor status are highly variable in human milk oligosaccharides from women delivering preterm. *Journal of Proteome Research*.

[15] Binia A., Samuel T. M., De Castro C. A. (2018). Human milk oligosaccharides profiles from healthy European mothers: New insights from Atlas of human milk nutrients, a multicenter observational cohort. ESPGHAN 51 Annual Meeting. *Journal of Pediatric Gastroenterology and Nutrition*.

[16] Smilowitz J. T., Totten S. M., Huang J. (2013). Human milk secretory immunoglobulin a and lactoferrin n-glycans are altered in women with gestational diabetes mellitus. *Journal of Nutrition*.

[17] Marcobal A., Barboza M., Sonnenburg E. D. (2011). Bacteroides in the infant gut consume milk oligosaccharides via mucus-utilization pathways. *Cell Host & Microbe*.

[18] Collado M. C., Cernada M., Baüerl C., Vento M., Pérez-Martínez G. (2014). Microbial ecology and host-microbiota interactions during early life stages. *Gut Microbes*.

[19] Yu Z.-T., Chen C., Newburg D. S. (2013). Utilization of major fucosylated and sialylated human milk oligosaccharides by isolated human gut microbes. *Glycobiology*.

[20] Sela D. A., Chapman J., Adeuya A. (2008). The genome sequence of Bifidobacterium longum subsp. infantis reveals adaptations for milk utilization within the infant microbiome. *Proceedings of the National Acadamy of Sciences of the United States of America*.

[21] Asakuma S., Hatakeyama E., Urashima T. (2011). Physiology of consumption of human milk oligosaccharides by infant gut-associated bifidobacteria. *The Journal of Biological Chemistry*.

[22] Marcobal A., Barboza M., Froehlich J. W. (2010). Consumption of human milk oligosaccharides by gut-related microbes. *Journal of Agricultural and Food Chemistry*.

[23] LoCascio R. G., Ninonuevo M. R., Freeman S. L. (2007). Glycoprofiling of bifidobacterial consumption of human milk oligosaccharides demonstrates strain specific, preferential consumption of small chain glycans secreted in early human lactation. *Journal of Agricultural and Food Chemistry*.

[24] Berger B., Grathwohl D., Alliet P., Puccio G., Steenhout P., Sprenger N. (2016). Stool microbiota in term infants fed formula supplemented with Human Milk Oligosaccharides and reduced likelihood of antibiotic use. *Journal of Pediatric Gastroenterology and Nutrition*.

[25] Steenhout P., Sperisen P., Martin F.-P. (2016). Term infant formula supplemented with human milk oligosaccharides (2'Fucosyllactose and Lacto-N-neotetraose) shifts stool microbiota and metabolic signatures closer to that of breastfed infants. Experimental Biology 2016 Meeting. *The FASEB Journal*.

[26] Lewis Z. T., Totten S. M., Smilowitz J. T. (2015). Maternal fucosyltransferase 2 status affects the gut bifidobacterial communities of breastfed infants. *Microbiome*.

[27] Smith-Brown P., Morrison M., Krause L., Davies P. S., Weir T. L. (2016). Mothers secretor status affects development of childrens microbiota composition and function: a pilot study. *PLoS ONE*.

[28] Newburg D. S., Ruiz-Palacios G. M., Morrow A. L. (2005). Human milk glycans protect infants against enteric pathogens. *Annual Review of Nutrition*.

[29] Newburg D. S., Ruiz-Palacios G. M., Altaye M. (2004). Innate protection conferred by fucosylated oligosaccharides of human milk against diarrhea in breastfed infants. *Glycobiology*.

[30] Morrow A. L., Ruiz-Palacios G. M., Altaye M. (2004). Human milk oligosaccharides are associated with protection against diarrhea in breast-fed infants. *Journal of Pediatrics*.

[31] Morozov V., Hansman G., Hanisch F., Schroten H., Kunz C. (2018). Human milk oligosaccharides as promising antivirals. *Molecular Nutrition & Food Research*.

[32] Martín-Sosa S., Martín M.-J., Hueso P. (2002). The sialylated fraction of milk oligosaccharides is partially responsible for binding to enterotoxigenic and uropathogenic Escherichia coli human strains. *Journal of Nutrition*.

[33] Lesman-Movshovich E., Lerrer B., Gilboa-Garber N. (2003). Blocking of *Pseudomonas aeruginosa* lectins by human milk glycans. *Canadian Journal of Microbiology*.

[34] Andersson B., Porras O., Hanson L. A., Lagergard T., Svanborg-Eden C. (1986). Inhibition of attachment of Streptococcus pneumoniae and Haemophilus influenzae by human milk and receptor oligosaccharides. *The Journal of Infectious Diseases*.

[35] Jantscher-Krenn E., Lauwaet T., Bliss L. A., Reed S. L., Gillin F. D., Bode L. (2012). Human milk oligosaccharides reduce *Entamoeba histolytica* attachment and cytotoxicity *in vitro*. *British Journal of Nutrition*.

[36] Ruiz-Palacios G. M., Cervantes L. E., Ramos P., Chavez-Munguia B., Newburg D. S. (2003). Campylobacter jejuni binds intestinal H(O) antigen (Fuc*α*1, 2Gal*β*1, 4GlcNAc), and fucosyloligosaccharides of human milk inhibit its binding and infection. *The Journal of Biological Chemistry*.

[37] Gustafsson A., Hultberg A., Sjöström R. (2006). Carbohydrate-dependent inhibition of Helicobacter pylori colonization using porcine milk. *Glycobiology*.

[38] Hong P., Ninonuevo M. R., Lee B., Lebrilla C., Bode L. (2009). Human milk oligosaccharides reduce HIV-1-gp120 binding to dendritic cell-specific ICAM3-grabbing non-integrin (DC-SIGN). *British Journal of Nutrition*.

[39] van Liempt E., Bank C. M. C., Mehta P. (2006). Specificity of DC-SIGN for mannose- and fucose-containing glycans. *FEBS Letters*.

[40] Naarding M. A., Ludwig I. S., Groot F. (2005). Lewis X component in human milk binds DC-SIGN and inhibits HIV-1 transfer to CD4^+^ T lymphocytes. *The Journal of Clinical Investigation*.

[41] Jantscher-Krenn E., Zherebtsov M., Nissan C. (2012). The human milk oligosaccharide disialyllacto-N-tetraose prevents necrotising enterocolitis in neonatal rats. *Gut*.

[42] Kuntz S., Rudloff S., Kunz C. (2008). Oligosaccharides from human milk influence growth-related characteristics of intestinally transformed and non-transformed intestinal cells. *British Journal of Nutrition*.

[43] Kuntz S., Kunz C., Rudloff S. (2009). Oligosaccharides from human milk induce growth arrest via G2/M by influencing growth-related cell cycle genes in intestinal epithelial cells. *British Journal of Nutrition*.

[44] Bode L., Rudloff S., Kunz C., Strobel S., Klein N. (2004). Human milk oligosaccharides reduce platelet-neutrophil complex formation leading to a decrease in neutrophil *β* 2 integrin expression. *Journal of Leukocyte Biology*.

[45] Bode L., Kunz C., Muhly-Reinholz M., Mayer K., Seeger W., Rudloff S. (2017). Inhibition of monocyte, lymphocyte, and neutrophil adhesion to endothelial cells by human milk oligosaccharides. *Thrombosis and Haemostasis*.

[46] Eiwegger T., Stahl B., Schmitt J. (2004). Human milk–derived oligosaccharides and plant-derived oligosaccharides stimulate cytokine production of cord blood t-cells in vitro. *Pediatric Research*.

[47] Eiwegger T., Stahl B., Haidl P. (2010). Prebiotic oligosaccharides: In vitro evidence for gastrointestinal epithelial transfer and immunomodulatory properties. *Pediatric Allergy and Immunology*.

[48] Goehring K. C., Marriage B. J., Oliver J. S., Wilder J. A., Barrett E. G., Buck R. H. (2016). Similar to those who are breastfed, infants fed a formula containing 2'-fucosyllactose have lower inflammatory cytokines in a randomized controlled trial. *Journal of Nutrition*.

[49] Sprenger N., Odenwald H., Kukkonen A. K., Kuitunen M., Savilahti E., Kunz C. (2017). FUT2-dependent breast milk oligosaccharides and allergy at 2 and 5 years of age in infants with high hereditary allergy risk. *European Journal of Nutrition*.

[50] Marriage B. J., Buck R. H., Goehring K. C., Oliver J. S., Williams J. A. (2015). Infants fed a lower calorie formula with 2 ′ fl show growth and 2 ′ fl uptake like breast-fed infants. *Journal of Pediatric Gastroenterology and Nutrition*.

[51] Puccio G., Alliet P., Cajozzo C. (2017). Effects of infant formula with human milk oligosaccharides on growth and morbidity. *Journal of Pediatric Gastroenterology and Nutrition*.

[52] Sprenger N., Lee L. Y., De Castro C. A., Steenhout P., Thakkar S. K. (2017). Longitudinal change of selected human milk oligosaccharides and association to infants' growth, an observatory, single center, longitudinal cohort study. *PLoS ONE*.

[53] Davis J. C., Lewis Z. T., Krishnan S. (2017). Growth and morbidity of gambian infants are influenced by maternal milk oligosaccharides and infant gut microbiota. *Scientific Reports*.

[54] Kuhn L., Kim H., Hsiao L. (2015). Oligosaccharide composition of breast milk influences survival of uninfected children born to hiv-infected mothers in lusaka, zambia. *Journal of Nutrition*.

